# Revisiting Whooping Cough: Global Drivers and Implications of Pertussis Resurgence in the Acellular Vaccine Era

**DOI:** 10.3390/vaccines14010035

**Published:** 2025-12-28

**Authors:** Siheng Zhang, Yan Xu, Ying Xiao

**Affiliations:** 1 Faculty of Medicine, Macau University of Science and Technology, Taipa, Macao SAR 999078, China; hengzsjnu@yeah.net; 2 Zhejiang Provincial Center for Disease Control and Prevention, Hangzhou 310051, China

**Keywords:** *Bordetella pertussis*, pertussis resurgence, acellular vaccine, vaccine-driven evolution, antimicrobial resistance, whole-genome sequencing

## Abstract

**Background**: Whooping cough caused by *Bordetella pertussis* is re-emerging despite high vaccination coverage, with rising incidence in adolescents and adults in the acellular vaccine (aP) era. This narrative review synthesizes evidence on the drivers of this paradox and their implications for pertussis control. **Methods**: We conducted a structured (but not fully systematic) literature search and narrative synthesis of PubMed, Web of Science, and Embase for publications from January 2000 to February 2025 using terms related to “*Bordetella pertussis*,” “pertussis resurgence,” “acellular vaccine,” “waning immunity,” “ptxP3,” “pertactin-deficient,” “macrolide resistance,” and “whole-genome sequencing.” English-language, peer-reviewed studies, surveillance reports, genomic analyses, and immunological investigations were included. About 1900 records met broad eligibility criteria and were screened, and key studies were selected for narrative synthesis. **Results**: The resurgence appears to result from three convergent factors: (1) waning and non-sterilizing aP-induced immunity, which allows bacterial colonization and transmission; (2) vaccine-driven genomic evolution of *B. pertussis*, marked by global dominance of the ptxP3 lineage and widespread pertactin-deficient (PRN−) strains; and (3) emergence of macrolide-resistant clones, exemplified by the MT28-Shanghai strain. Whole-genome sequencing (WGS) has been central for defining these processes and clonal sweeps under combined vaccine and antibiotic pressure, supporting a three-driver framework of waning aP immunity, vaccine-driven evolution, and macrolide resistance. **Conclusions**: Pertussis resurgence illustrates pathogen adaptation to human interventions. Effective mitigation requires WGS-integrated global surveillance, re-evaluation of vaccine formulations to keep pace with antigenic change, and strengthened antibiotic stewardship, alongside development of next-generation vaccines that induce durable mucosal immunity and block transmission.

## 1. Background

**Figure 1:**
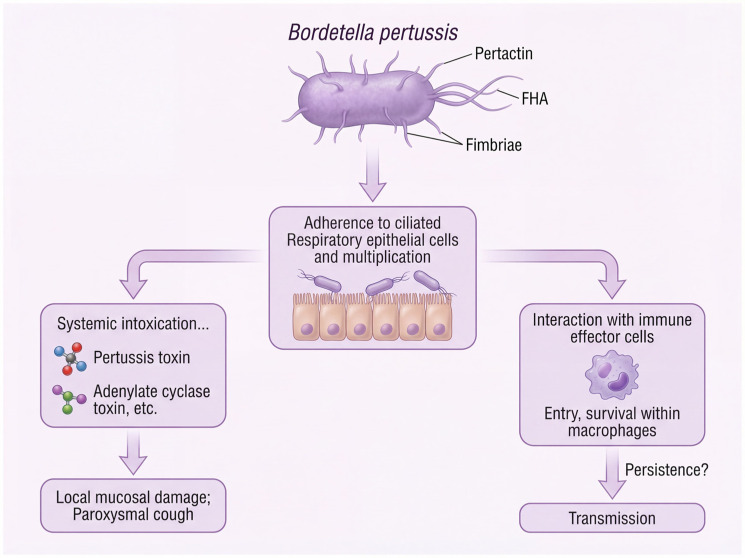
Key stages in the pathogenesis of *Bordetella pertussis* infection. The schematic depicts whooping cough progression from bacterial attachment to ciliated respiratory epithelium via adhesins (Pertactin, FHA and fimbriae), through mucosal colonization and replication, to toxin-mediated damage. Pertussis toxin causes systemic intoxication, whereas adenylate cyclase toxin, tracheal cytotoxin and dermonecrotic toxin injure the airway locally, driving paroxysmal cough and impaired mucociliary clearance. In parallel, interaction with macrophages allows immune modulation and persistence. Together, these processes underpin effective colonization and onward transmission.

Whooping cough, also known as pertussis, is an extremely contagious respiratory disease which is caused by the bacterium *Bordetella pertussis* [1]. It is envisaged that the typical clinical presentation of paroxysmal coughing followed by an inspiratory whoop and post-tussive vomiting is a result of a sophisticated multi-stage pathogenesis. As shown in Figure 1, infection initiates with the adherence of the bacteria to ciliated respiratory epithelium through important adhesins such as Pertactin (PRN), Filamentous Hemagglutinin (FHA) and Fimbriae (FIM). The later local multiplication and activity of contributing toxins, among others Pertussis Toxin (PT), Adenylate Cyclase Toxin (ACT) and Tracheal Cytotoxin (TCT), cause deep mucosal disruption, loss of ciliary clearance but also erythrogenic systemic intoxication resulting in characteristic clinical manifestations [2]. This well-defined pathological process made pertussis an excellent target for a vaccine.

For years, whooping cough was lauded as a public health success story. Whole-cell pertussis (wP) vaccines introduced in the mid-20th century led to a substantial reduction in the incidence of this disease, which can be particularly severe and fatal in infants. Yet since the 1990s, with increasing replacement of wP vaccines by less reactogenic acellular (aP) vaccines in many high-income countries, a disturbing new epidemiological trend has appeared: pertussis is resurging despite still high vaccination coverage [3]. This resurgence is starkly illustrated by the 2012 outbreak in Washington state, where reported cases soared to 2520-a 1300% increase from the same period in 2011. The outbreak revealed critical vulnerabilities, with the highest rates among infants under one year, 10-year-old children, and surprisingly, adolescents who had been fully vaccinated with Tdap. Furthermore, significant disparities emerged, as the incidence among Hispanics was more than twice that of non-Hispanics (53.1 vs. 24.6 cases per 100,000 population) [4]. This ‘pertussis paradox’ has prompted a critical re-evaluation of current control strategies.

The central argument of this narrative review is that the resurgence is driven by a confluence of factors rooted in the acellular vaccine era. The shift to aP vaccines, while reducing adverse reactions, introduced new ecological pressures that have shaped both host immunity and the pathogen itself. The core drivers are threefold: (1) the imperfect and waning nature of aP immunity; (2) the rapid, vaccine-driven genomic evolution of *B. pertussis*; and (3) the selective pressure of antimicrobial use, leading to resistance, which acts as a compounding threat. The implications of these drivers are profound, altering the epidemiology of the disease, challenging the efficacy of existing medical countermeasures, and demanding a re-evaluation of global public health approaches. This review proposes a three-driver framework integrating waning aP immunity, vaccine-driven genomic evolution, and emerging macrolide resistance to explain pertussis resurgence, and highlights Whole-genome sequencing (WGS)-informed surveillance as a central pillar for future control. Evidence supporting each driver is reviewed in detail in Section 4, Section 5 and Section 6 [2,3].

This narrative review revisits the whooping cough story by first dissecting these primary drivers. It then explores the consequential shifts in the epidemiological landscape and concludes with a discussion of the necessary future directions for research, surveillance, and vaccine development to regain control over this resilient pathogen.

## 2. Methodology

### 2.1. Search Strategy and Study Selection

This narrative review aimed to synthesize contemporary evidence on the drivers and public health implications of pertussis resurgence in the acellular vaccine era. We performed a structured literature search in PubMed, Web of Science Core Collection and Embase for articles published between January 2000 and February 2025. Additional records identified via PubMed and Google Scholar were used to complement background coverage and citation chaining, but were not systematically counted in the flow diagram.

We combined the following keywords and MeSH terms in various Boolean strings: “*Bordetella pertussis*”, “pertussis resurgence”, “acellular vaccine”, “waning immunity”, “ptxP3”, “pertactin-deficient”, “macrolide resistance”, “whole genome sequencing”, and “WGS surveillance”.

Basic eligibility criteria were predefined as follows: (i) English-language publications between 2000 and February 2025; (ii) primary studies or surveillance reports presenting original epidemiological, immunological, or genomic data on human pertussis; and (iii) content directly informing at least one of the three drivers (waning/imperfect aP immunity, vaccine-driven genomic evolution, or antimicrobial resistance). We excluded editorials, commentaries, conference abstracts, and studies lacking primary data or clear relevance to the review question.

**Figure 2:**
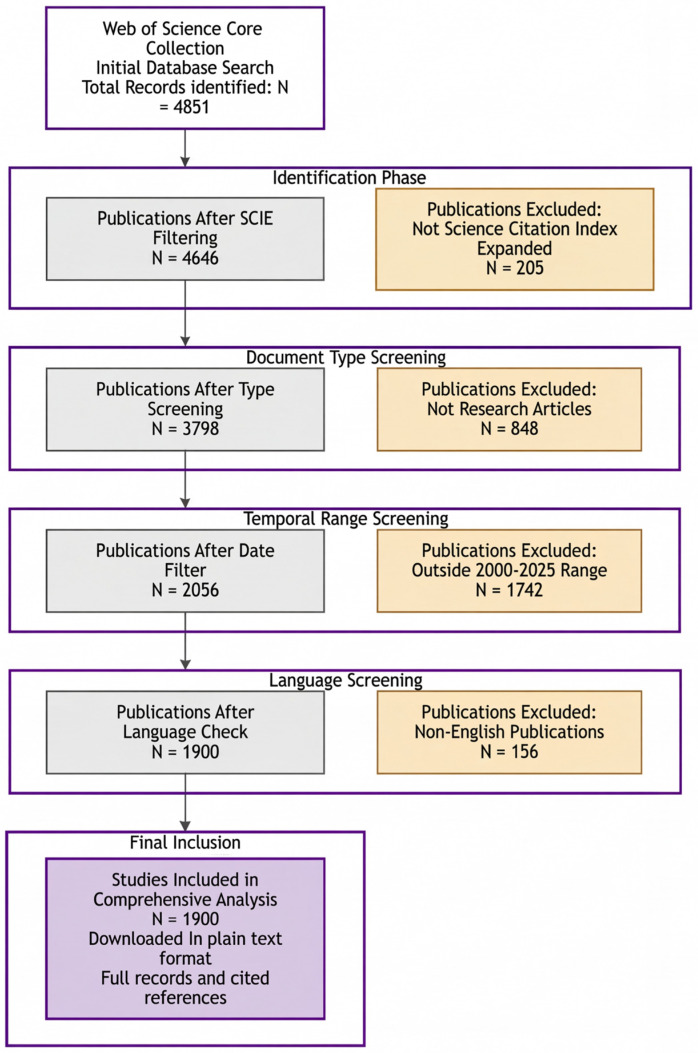
Flow diagram of the literature search and study selection for this narrative review. The flow diagram summarizes the structured process of literature search and study selection. After sequential filters and duplicate removal, 1900 records met the basic eligibility criteria and were screened, from which key studies were selected for detailed narrative synthesis.

From an initial yield of 4851 records in Web of Science and Embase, as shown in Figure 2, we applied sequential filters based on index coverage, document type, publication year, and language. After removing duplicates across databases, 1900 publications met the basic eligibility criteria and were screened. Given the narrative and framework-building scope of this review, we did not pre-register a protocol, aim for exhaustive coverage, or conduct a formal risk of bias assessment. Instead, we selected key studies judged to be methodologically robust and highly relevant (e.g., well-described study design, adequate sampling and case definitions, and/or high-resolution genomic methods such as WGS) to construct a coherent narrative synthesis around the three core drivers framework.

### 2.2. Data Extraction and Synthesis

For each included study we extracted: (i) first author and year; (ii) study design and geographical setting; (iii) evidence relevant to the three core drivers (waning acellular vaccine immunity, pathogen evolution, antimicrobial resistance); (iv) genomic methodologies (such as WGS, multilocus variable-number tandem-repeat analysis (MLVA)); and (v) key public health implications. Extracted findings were grouped thematically into immunology, genomics, epidemiology and antimicrobial resistance, and integrated into a three-driver framework to explain pertussis resurgence. Data were synthesized narratively rather than pooled quantitatively, with emphasis on convergent patterns and clinically or policy-relevant signals.

## 3. Clinical Presentation and Host Immunity

*Bordetella pertussis*, a Gram-negative coccobacillus discovered in 1906, is an obligate human pathogen with a multilevel virulence strategy. Traditionally considered non-motile, it has more recently been demonstrated that *B. pertussis* can produce a flagellum-like structure during infection that may aid further spread and transmission [5]. The virulence of the pathogen is mediated by a capsule, which provides antiphagocytic properties, pili that facilitate adhesion and an elaborate arsenal of toxins and adhesins [6]. These virulence factors play a crucial role in its pathogenesis and thus shape host immunity.

The disease process is initiated by inhalation of *B. pertussis* from respiratory droplets. The bacterium attaches specifically to human respiratory tract ciliated epithelial cells by means of adhesins, predominantly filamentous hemagglutinin (FHA), fimbriae (FIM) and pertactin (PRN) [6]. Upon successful attachment, the bacterium locally replicates and produces diverse toxins. One of the typical AB_5_ exotoxins from *B. pertussis* is Pertussis toxin (PT), which plays a key role in both virulence and protective immunity [7,8]. PT inhibits intracellular signaling pathways, leading to an unregulated accumulation of cyclic adenosine monophosphate (cAMP) and thereby affecting immune cell activity [9,10]. This disturbance results in reduced neutrophil and macrophage recruitment, with a resulting profound lymphocytosis, which is typical. Furthermore, local mucosal damage is enhanced by the action of ACT and TCT which intoxify immune cells and destroy ciliated epithelium, respectively.

**Figure 3:**
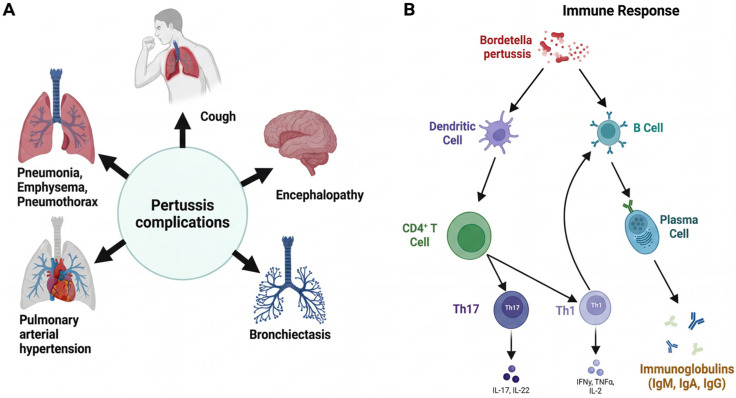
Complications of disease and adaptive immune responses in pertussis. (**A**) The paroxysmal cough, which is characteristic of whooping cough, can lead to a variety of complications such as respiratory sequelae (e.g., pneumonia, pneumothorax, bronchiectasis and pulmonary arterial hypertension) and neurological impairments including encephalopathy. A characteristic hematological feature of the disease is lymphocytosis. (**B**) Adaptive immune response after *Bordetella pertussis* infection. This involves the stimulation of B cells and a key CD4^+^ T-cell response. Efficient and protective immune response is characterized by the Th1 (IFN-γ and IL-2 producer) and Th17 (IL-17 producer) differentiation from T-helper subsets, which are essential to orchestrate bacterial clearance from the respiratory system.

This pathophysiology underlies the serious complications of pertussis, which are both respiratory-induced (atelectasis, emphysema, bronchiectasis and pneumonia) as well as being due to injury to the central nervous system from hypoxia (pertussis encephalopathy). Extensive leukocytosis also contributes to pulmonary hypertension, a major complication [11]. A scheme for these complications is shown in Figure 3A.

Protection against *B. pertussis* requires a coordinated humoral and cellular response within the adaptive immune system. While antibodies are critical for disease protection, Cluster of Differentiation 4-positive (CD4^+^) T cell-mediated responses are required for bacterial clearance and the development of long-term immunity [12,13]. As depicted in Figure 3B, an optimal immune response is associated with induction of T-helper 1 (Th1) and T-helper 17 (Th17) cells. Th1 cells are essential for opsonizing antibodies, and for activating cell-mediated immunity via Interferon-γ (IFN-γ), Interleukin-2 (IL-2), and Interleukin-12 (IL-12) production. Th17 cells, which secrete Interleukin-17 (IL-17), are critical for the recruitment of neutrophils and for mucosal immunity with resident-memory T cells in the airways providing additional protection against reinfection [14]. This immune landscape, particularly the Th1/Th17 response, forms the critical foundation for understanding the failures of acellular vaccine-induced immunity discussed in the next section.

PT, FIM and PRN antigens all elicit production of protective antibodies. PT-Immunoglobulin G (IgG) serology is a gold standard for diagnosis, epidemiological study and immunity assessment due to its high and specific immunogenicity [15]. While PT is well-known to be important in induction of antibody, PT can also increase cell-mediated immunity by altering cytokine production and directly activating CD4^+^ T cells [16]. Finally, the multifaceted immune response is the key for a successful clearance of *B. pertussis* infection and defects in its components, famously those arising from acellular vaccine-mediated immunity, underlie the reemergence of the disease as shown in subsequent sections.

## 4. The Primary Drivers of Resurgence

### 4.1. Driver 1: Waning and Imperfect Acellular Vaccine Immunity

The first major driver of resurgence is the nature of the immunity conferred by aP vaccines. While effective at preventing severe disease, aP vaccines differ critically from wP vaccines in the type and duration of protection they elicit. Unlike whole-cell vaccines, which induce a broad Th1-mediated immune response, aP vaccines prime a Th2-dominated response, which is less effective at preventing bacterial colonization and transmission [17]. Direct comparative studies show that wP vaccination induces significantly higher levels of Th1 (IFN-γ) and Th17 (IL-17) cytokines and mucosal Immunoglobulin A (IgA), which are crucial for bacterial clearance at the respiratory mucosa [18]. This fundamental difference in the immune response means that aP-vaccinated individuals can become asymptomatically colonized and transmit the bacteria, unknowingly acting as reservoirs in the community. The cellular immunity elicited by wP vaccines, particularly Th1 and Th17 responses, is more effective at clearing the bacteria from the respiratory tract, thereby reducing carriage and transmission. The aP vaccine’s reliance on a Th2-skewed response, characterized by high antibody titers but less robust T-cell mediated immunity, creates a permissive environment for silent circulation of the pathogen.

Furthermore, numerous studies have demonstrated that aP-induced immunity wanes relatively quickly [19,20,21]. Notably, the relative durability of immunity after aP versus wP vaccination remains debated. While multiple cohort and effectiveness studies support substantial waning following aP schedules, direct comparisons are complicated by heterogeneity in historical wP formulations, differences in study design, endpoints (infection vs. disease), and background exposure. Recent syntheses have emphasized that waning is not unique to aP and that the magnitude of any difference between aP and wP may vary across settings and vaccine products. This uncertainty is important when interpreting “waning” as a driver and when considering future vaccine development and policy options [22]. Protection against disease begins to decline significantly as early as 3–5 years post-vaccination, leaving older children, adolescents, and adults susceptible to symptomatic infection [19]. This creates a large pool of susceptible individuals who can fuel transmission cycles and expose vulnerable, unvaccinated infants, for whom the disease remains most dangerous. The reliance on a vaccine that does not provide durable, sterilizing immunity is a fundamental limitation that the pathogen has exploited. Epidemiological models consistently show that the switch to aP vaccines, with their shorter duration of protection and inability to prevent transmission, is a key factor explaining the re-emergence of pertussis in highly vaccinated populations. The combination of waning antibody levels and the lack of robust mucosal immunity creates an immune landscape that is permissive for the persistence and spread of *B. pertussis* [23]. Thus, aP vaccines primarily prevent severe disease but allow subclinical carriage and transmission, a central flaw exposed by the genomic and epidemiological data reviewed in subsequent sections.

### 4.2. Driver 2: Vaccine-Driven Pathogen Evolution

The second, and perhaps most significant, driver is the rapid evolution of *B. pertussis* in response to the selective pressure exerted by aP vaccines. The aP formulations contain a limited set of antigens (typically PT, PRN, FHA, and FIM), creating a strong incentive for the bacterium to alter or discard these targeted components [24]. This process of vaccine-driven antigenic divergence is a powerful example of contemporary pathogen adaptation.

The most striking evolutionary success story is the ptxP3 lineage, which has replaced the ancestral ptxP1 lineage worldwide and is associated with enhanced pertussis toxin production, potentially increasing transmission fitness and suppressing host immunity [25,26]. The global sweep of ptxP3 strains is a clear signature of vaccine-driven selection. The ptxP3 allele features a mutation in the promoter region of the pertussis toxin gene, leading to increased PT production. This elevated toxin output may enhance the bacterium’s ability to suppress host immune responses, facilitate colonization, and promote transmission, providing a distinct advantage in populations where immunity to the toxin is common due to vaccination [27]. The emergence and dominance of ptxP3 have been documented across continents, from Europe and North America to Asia and Australia, always following the widespread introduction of aP vaccines [28]. The acellular vaccine preparations target a limited number of critical virulence factors, principally adhesins and pertussis toxin, which exert direct selective pressure towards their modification. The structure, function and vaccine status of these and other major virulence factors are listed in Table 1.

Concurrently, there has been a convergent, global emergence of strains that lack PRN, a key vaccine component. Pertactin-deficient (PRN-) strains now comprise the majority of circulating isolates in many countries, as the loss of this antigen allows the bacterium to evade PRN-specific antibodies without a significant fitness cost [24,29]. Independent PRN-disrupting events in multiple regions suggest strong, convergent vaccine-driven selection rather than genetic drift. This represents a direct adaptation to aP-induced immunity. The mechanisms for PRN deficiency are diverse, including insertions of mobile genetic elements (IS481), point mutations introducing early stop codons, and large-scale gene deletions. The fact that these independent mutational events have arisen and swept through populations in different geographic regions simultaneously is a powerful testament to the strength of vaccine selection. In vitro studies and animal models have confirmed that PRN-strains are not attenuated in their ability to colonize hosts but are effectively shielded from anti-PRN antibody-mediated killing.

Antigenic divergence is also observed in other vaccine antigens, such as fimbrial proteins (FIM2 and FIM3) and the pertussis toxin subunit A (PtxA), further widening the gap between vaccine strains and circulating populations. The accumulation of these changes in the ptxP3 genetic background has created a globally successful, vaccine-adapted clone that is antigenically distinct from the strains used to manufacture aP vaccines [30].

**Table 1:** The *Bordetella pertussis* virulome: targets of acellular vaccines and documented evolutionary escape mechanisms.

Virulence Factor [Reference]	Structure & Location	Role in Pathogenesis	Included in aP Vaccines?	Evolution & Vaccine Evasion
Toxins				
Pertussis Toxin (PT) [31]	Secreted AB_5_-type exotoxin	Ribosylates inhibitory G proteins, disrupting cellular signaling and causing systemic symptoms (e.g., lymphocytosis).	Yes (All formulations)	ptxP3 lineage: Promoter mutation increases PT production, potentially enhancing transmission fitness and immune suppression [27,28]
Adenylate Cyclase Toxin (ACT) [32]	RTX toxin (Extracytoplasmic)	Converts intracellular ATP to cAMP, disabling immune effector cells (phagocytes).	No	Not under direct vaccine pressure, but its role in neutralizing innate immunity remains critical for colonization.
Tracheal Cytotoxin (TCT) [33]	Peptidoglycan fragment (Extracellular)	Damages ciliated respiratory cells, inhibiting mucociliary clearance.	No	Evolution not documented; its effect is synergistic with other toxins.
Dermonecrotic Toxin (DNT) [34]	Heat-labile A-B toxin (Cytoplasm)	Activates Rho GTPase, causing vasoconstriction and cell death.	No	Not a target of vaccine-induced immunity.
Lipo-oligosaccharide (LOS) [35]	Endotoxin (Surface)	Triggers pro-inflammatory responses; contributes to coughing.	No	Not a target of vaccine-induced immunity.
Adhesins				
Pertactin (PRN) [24,29]	Autotransporter protein (Surface)	Mediates binding to host cells; confers resistance to neutrophil-mediated clearance.	Yes (3- & 5-component)	Pertactin-deficiency (PRN-): Widespread global emergence via IS481 insertions, point mutations, and deletions, allowing evasion of PRN-specific antibodies [36].
Fimbriae (FIM2/3) [30,37]	Filamentous proteins (Surface)	Facilitate attachment to tracheal epithelial cells; serotype-specific immunity.	Yes (FIM2/3 in multi-component)	Antigenic Divergence: Amino acid changes in FIM2 and FIM3 proteins reduce antibody recognition, widening the antigenic gap [38,39].
Filamentous Hemagglutinin (FHA) [40]	Filamentous protein (Cell wall)	Key adhesin; binds to ciliated epithelium and immune cell receptors.	Yes (Most formulations)	Antigenic variation is less common than for PRN or FIM, but its role in attachment remains vital.
Other systems				
Type III Secretion System (T3SS) [41,42]	Needle-like injectisome (Cell envelope)	Injects effector proteins directly into host cells.	No	A potential target for next-generation vaccines, as it is essential for virulence and conserved.

### 4.3. Driver 3: The Emergence of Antimicrobial Resistance

The third driver, concentrated regionally but with global implications, is the emergence of macrolide-resistant *B. pertussis*. This driver represents a compounding threat, demonstrating that the pathogen is evolving under dual selective pressures from both vaccines and antibiotics. Macrolide antibiotics like azithromycin are the first-line treatment for pertussis and are critical for post-exposure prophylaxis to prevent outbreaks, especially in healthcare settings and households with vulnerable infants.

**Figure 4:**
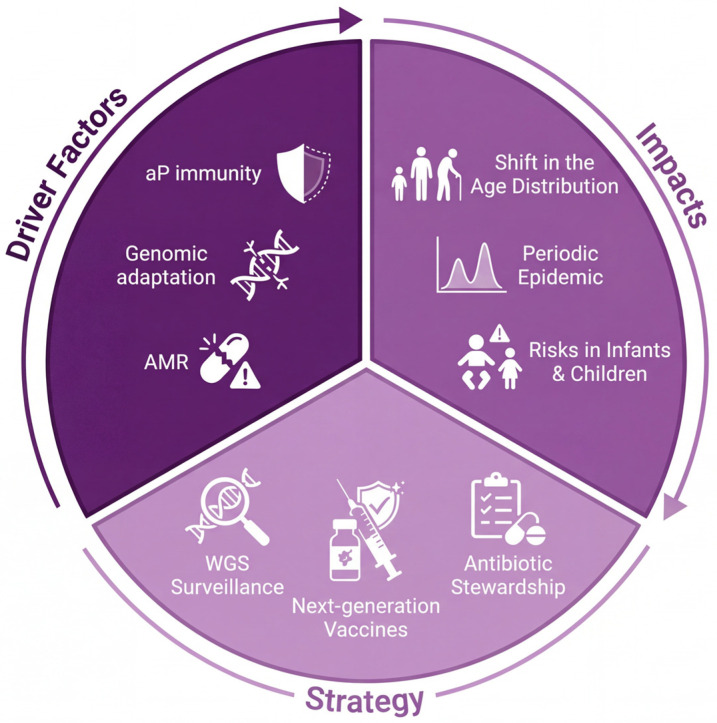
Integrative model of pertussis resurgence in the acellular vaccine era. The schematic illustrates the triad of primary drivers (waning aP immunity, pathogen genomic adaptation, and antimicrobial resistance), their convergent epidemiological impacts, and the essential public health interventions required for mitigation.

In China, and sporadically elsewhere in Asia, the MT28-Shanghai clone, a ptxP3 strain carrying the A2047G mutation in the *23S rRNA* gene, has become prevalent, conferring high-level resistance to macrolides [43,44,45]. This clone is a quintessential example of convergent adaptation, possessing key vaccine-evading mutations (ptxP3, PRN-) alongside treatment-evading resistance. The emergence and spread of this clone are directly linked to the high volume of macrolide prescriptions for respiratory infections in the region. The A2047G mutation alters the drug-binding site on the bacterial ribosome, reducing the affinity of macrolide antibiotics and rendering standard treatments ineffective. This resistance threatens to undermine a cornerstone of pertussis management, potentially prolonging infectiousness and complicating outbreak control [46]. Details on the mechanisms and molecular epidemiology of this resistance are discussed in Section 6. The potential for international spread through travel makes this a concern for the global community. While alternative antibiotics like trimethoprim-sulfamethoxazole exist, their use is not ideal, particularly in young infants, due to potential safety concerns. The convergence of macrolide resistance with vaccine-adaptive mutations in a single, successful clone exemplifies the pathogen’s remarkable ability to evolve under multiple, simultaneous selective pressures, posing a multifaceted challenge to disease control. Figure 4 provides a conceptual overview of how these three primary drivers collectively lead to common epidemiological impacts, underscoring the necessity for integrated interventions centered on genomic surveillance, next-generation vaccines, and antibiotic stewardship.

## 5. Genomic Epidemiology and Global Surveillance

### 5.1. Geographic Distribution of Lineages

Although genetically monomorphic at the whole-genome level, *B. pertussis* exhibits marked regional lineage structures, largely shaped by local vaccination and antibiotic-use patterns. Over the past two decades, comparative genomic studies have revealed striking geographic heterogeneity in the population structure of *Bordetella pertussis*. Subtle lineage differences, particularly in key regulatory and antigenic loci, have resulted in distinct regional profiles. Among these, the ptxP3 lineage has emerged as the globally dominant strain type, gradually replacing the ancestral ptxP1 lineage in most countries where aP vaccines are routinely used [37]. This worldwide lineage shift reflects the combined influence of vaccine-driven selection, antibiotic use patterns, and population immunity.

The ptxP3 lineage was first identified in the Netherlands in the late 1990s during a resurgence of pertussis cases despite high vaccine coverage. Subsequent genomic analyses demonstrated that ptxP3 strains produced higher levels of pertussis toxin and exhibited enhanced transmission potential compared to their ptxP1 predecessors [47]. Within a few years, the lineage spread across Western Europe, and by the mid-2000s, ptxP3 isolates had become predominant in many European nations including the United Kingdom, France, Norway, and Italy, coinciding with large-scale adoption of aP vaccines. A similar replacement was documented in North America, where U.S. surveillance data show that by 2012, nearly all circulating isolates carried the ptxP3 promoter. Most of these also displayed pertactin deficiency (PRN-) due to gene disruption by IS481 insertions or deletions, reflecting adaptation under acellular vaccine pressure [24,29]. Notably, these North American and European lineages remained susceptible to macrolide antibiotics, suggesting that selection in these regions was driven primarily by immune rather than antimicrobial pressure.

In Asia, however, the evolutionary trajectory of *B. pertussis* has taken a more complex course. Genomic surveillance in China, Japan, and South Korea indicates that the ptxP3 lineage dominates there as well, but it has diversified into several subclonal branches, including those exhibiting macrolide resistance [28,48,49,50]. The most prominent among these is the MT28-Shanghai clone, which emerged in eastern China around 2013 and has since spread to multiple provinces. Epidemiological studies indicate that MT28-Shanghai is now the predominant macrolide-non-susceptible lineage in several high-incidence, high-antibiotic-use regions of eastern China, highlighting its successful regional expansion [51,52,53]. Its prevalence, exceeding 70% in some Chinese provinces, underscores the strong selective impact of widespread antibiotic use. Detailed mechanisms and clinical implications of macrolide resistance are discussed in Section 6. By contrast, Japanese isolates remain mostly macrolide-sensitive but show high rates of pertactin deficiency and unique allelic variants in fim2 and fim3, reflecting local evolutionary pressures associated with Japan’s long-standing booster vaccination policies [54,55].

In Australia and New Zealand, genomic monitoring has also documented rapid turnover of lineages. Following major outbreaks in 2008–2012, ptxP3 strains-often PRN-deficient-replaced historical ptxP1 populations, paralleling trends seen in Europe [56,57,58,59]. Interestingly, Australian isolates show close phylogenetic relatedness to those from the United States and Europe, suggesting frequent intercontinental exchange, possibly facilitated by international travel and migration. This observation reinforces the concept that *B. pertussis* evolution, though largely clonal, is interconnected globally through human movement [60,61].

Regions that continue to rely on wP vaccines, particularly in South America, Africa, and parts of Southeast Asia, display a somewhat different lineage composition, underscoring the global heterogeneity in *B. pertussis* populations. In these settings, ptxP1 strains remain more prevalent and strain diversity is higher, which may be attributed to the broader immune pressure exerted by wP vaccines. However, due to limited genomic data from these regions, our understanding might underestimate the ongoing penetration of the vaccine-adapted ptxP3 lineage [28,62]. For instance, surveillance in Brazil and certain African nations indicates a slower rate of lineage turnover compared to aP-using countries. Because whole-cell vaccines elicit broader immune responses against multiple antigens, they may impose less focused selective pressure on antigenic loci such as ptxP, PRN, or fim3. Consequently, these regions act as reservoirs of greater strain diversity. However, limited genomic data from these regions hinder a comprehensive assessment, highlighting a critical gap in our global understanding and the need for expanded sampling to track the incursion of vaccine-adapted ptxP3 strains.

**Figure 5:**
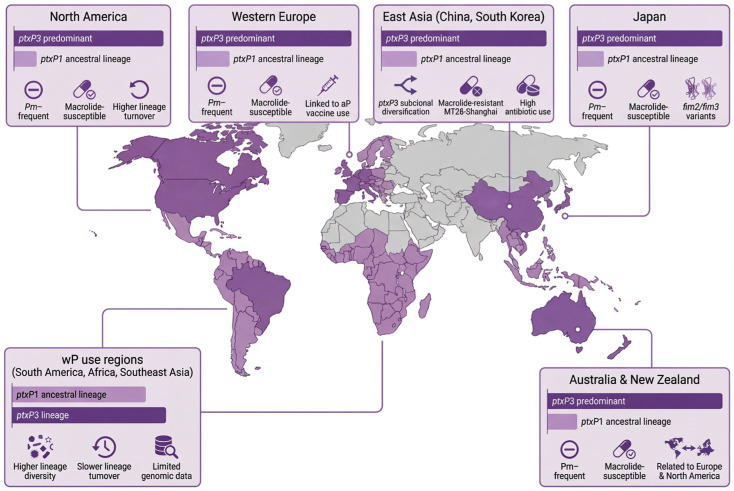
Geographic distribution of *Bordetella pertussis* lineages by major world regions (genomic surveillance). The schematic map displays dominant lineages in broad regions rather than individual countries. For each region, horizontal bars indicate the relative contribution of ptxP3 versus ptxP1 and other ancestral lineages, while short text labels highlight key features such as pertactin deficiency, macrolide resistance or increased lineage diversity. aP-using regions cluster toward ptxP3 predominance, whereas wP-using regions retain more heterogeneous populations. Symbol size reflects the relative importance of specific lineages within each region.

Regional genomic data indicate that the population structure of *B. pertussis* is now strongly stratified by vaccination strategy and antibiotic use. In aP-using countries of North America, Western Europe, Australia and New Zealand, ptxP3 lineages predominate, often with pertactin deficiency. East Asian settings combine ptxP3 dominance with macrolide-resistant sublineages such as MT28-Shanghai, whereas Japan shows ptxP3 with distinctive fim2/fim3 variants but largely preserved macrolide susceptibility. Regions relying mainly on wP vaccines in South America, Africa and parts of Southeast Asia retain more ptxP1 and higher lineage diversity. These contrasts are summarized in Figure 5.

Taken together, these findings emphasize that *B. pertussis* evolution is not uniform but reflects regional ecological niches created by public-health interventions. Understanding these geographic differences is essential for tailoring vaccination strategies, improving molecular surveillance, and anticipating future lineage dynamics. Continuous genomic monitoring-especially in underrepresented low- and middle-income countries-will be critical for building a complete picture of the global pertussis landscape and ensuring timely responses to emerging variants [63].

### 5.2. Whole-Genome Sequencing as a Surveillance Tool

Accurate surveillance of *Bordetella pertussis* is critical for understanding its epidemiology, detecting emerging lineages, and guiding public health interventions. Historically, traditional molecular typing methods such as MLVA and pulsed-field gel electrophoresis (PFGE) were the primary tools for strain characterization. While these approaches provided useful information on short-term outbreak dynamics and broad population structures, they have limited resolution, particularly when discriminating between closely related strains or assessing fine-scale evolutionary changes [64]. Additionally, MLVA and PFGE are unable to detect subtle genetic variations, such as single-nucleotide polymorphisms (SNPs), small insertions or deletions, or regulatory mutations in promoter regions, all of which can have significant effects on virulence, antigenicity, and transmissibility.

**Figure 6:**
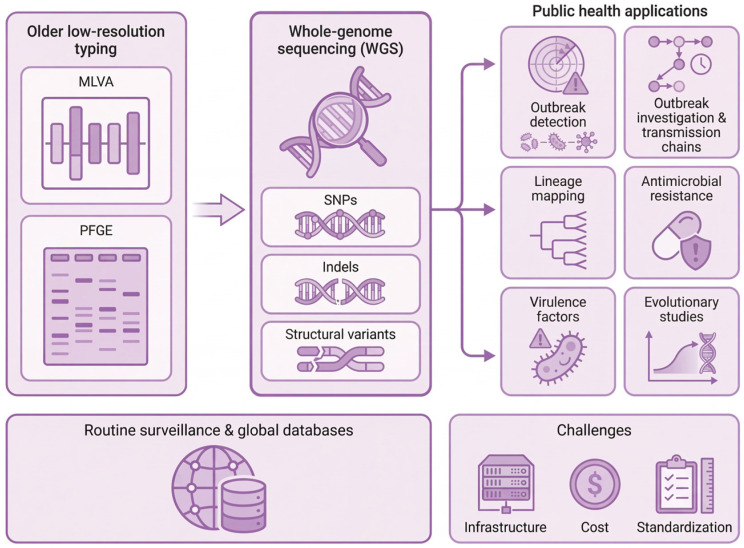
Utility of WGS for modern pertussis surveillance. The schematic contrasts older, low-resolution typing methods (MLVA and PFGE) with genome-wide WGS. While MLVA and PFGE provide limited discrimination, WGS delivers high-resolution coverage of single-nucleotide polymorphisms, indels and structural variants. These data support key public health functions, including precise outbreak detection and investigation, reconstruction of transmission chains, lineage mapping, identification of antimicrobial resistance and virulence factors, and long-term evolutionary analyses. The ultimate goal is integration of WGS into routine surveillance systems and global databases, although infrastructure, cost and standardization barriers persist.

More recently, the arrival of WGS has revolutionized pertussis surveillance by overtaking conventional low-resolution methods such as MLVA and PFGE and enabling high-resolution phylogenetic studies. As shown in Figure 6, WGS provides a comprehensive view of the pathogen genome, allowing identification of SNPs across the chromosome, tracking of adaptive mutations, and detection of structural variations in antigenic or regulatory genes. Its utility is demonstrated in several key public health applications: (1) distinguishing local from imported outbreaks; (2) enabling early detection of emerging antimicrobial resistance (AMR) clones; and (3) dynamically tracking the sweep of adaptive variants like ptxP3 and PRN-strains. This level of resolution is critical for proper lineage discrimination—including differentiation between the globally dominant ptxP3 lineage and regional subclones such as the MT28-Shanghai macrolide-resistant strain in China—determination of antigenic variants such as pertactin-deficient and fimbrial-altered strains, and identification of key resistance markers [59,65].

One of the key advantages of WGS is its ability to trace outbreaks in near real-time. Traditional surveillance often relies on clinical case reporting, which may lag behind actual transmission events. By integrating genomic data with temporal and spatial epidemiological information, public health authorities can reconstruct transmission networks, identify infection sources, and distinguish between locally circulating strains and imported variants [63]. For instance, WGS analyses in the United States and Europe have demonstrated that multiple co-circulating ptxP3 sublineages contribute simultaneously to epidemic peaks, a level of resolution impossible with older typing methods. This information can inform targeted vaccination campaigns and containment strategies.

Moreover, WGS enables the identification of virulence-associated mutations and antimicrobial resistance markers. The A2047G mutation in the *23S rRNA* gene, which confers high-level macrolide resistance, is readily detected through sequencing, facilitating early recognition of resistant lineages. Similarly, disruptions in the PRN gene, often caused by insertion sequences such as IS481 or IS1002, can be pinpointed at single-nucleotide resolution, allowing researchers to monitor the global emergence of pertactin-deficient strains [24,66]. By linking these molecular findings to epidemiological data, scientists can assess the fitness advantages of specific genotypes and anticipate shifts in the circulating population.

Beyond outbreak response, WGS provides a powerful tool for long-term evolutionary surveillance. Time-resolved phylogenetic analyses can estimate the rate of genomic change, track selective sweeps, and detect patterns of global lineage replacement. For example, studies using WGS have revealed the progressive replacement of ptxP1 by ptxP3 strains across multiple continents, as well as the independent emergence of pertactin-deficient clones in geographically distinct regions [67]. These insights highlight the ongoing adaptive evolution of *B. pertussis* under the combined pressures of vaccination and antimicrobial use.

The integration of WGS into routine national and international surveillance systems is becoming increasingly feasible due to reductions in sequencing costs, improvements in bioinformatics pipelines, and the establishment of centralized genomic databases [68]. Platforms such as the National Center for Biotechnology Information (NCBI) Pathogen Detection database and regional repositories allow real-time sharing and comparison of genomic sequences across countries, supporting global monitoring of emerging lineages and resistance trends. Such integration not only enhances outbreak preparedness but also informs vaccine policy decisions, including the potential need for updated vaccine strains or booster recommendations.

Despite its transformative potential, there are challenges to implementing WGS-based surveillance universally. Many low- and middle-income countries lack the infrastructure, sequencing capacity, and bioinformatics expertise required for routine genome sequencing. Additionally, standardization of protocols and quality control measures is essential to ensure comparability of data across laboratories and regions. Ethical considerations, including patient privacy and data-sharing agreements, must also be addressed to maximize the public health utility of genomic data while protecting individual rights. These challenges are particularly acute in low- and middle-income countries (LMICs), where pertussis burden may be underestimated but genomic data remain scarce.

## 6. Emergence of Antimicrobial Resistance

The use of antibiotics, particularly macrolides, has been central to the clinical management and prophylaxis of pertussis. While pertussis infections are often self-limiting in older children and adults, macrolides such as erythromycin, azithromycin, and clarithromycin are routinely prescribed to reduce symptom duration, limit transmission, and protect vulnerable infants [69,70]. For decades, *Bordetella pertussis* has been largely susceptible to macrolides, making them the first-line therapy worldwide. However, in recent years, the emergence of macrolide-resistant isolates has raised significant public health concerns, particularly in Asia, where selective pressures from widespread antibiotic use have facilitated the expansion of resistant clones.

### 6.1. Mechanisms of Macrolide Resistance

Macrolide resistance in *B. pertussis* is primarily conferred by a point mutation in the *23S ribosomal RNA* gene (A2047G, numbering based on E. coli 23S rRNA). This single-nucleotide polymorphism alters the macrolide binding site within the 50S ribosomal subunit, reducing drug affinity and resulting in high-level resistance [71,72]. Notably, this mutation does not appear to compromise bacterial fitness or virulence, a finding supported by both animal models and the successful global spread of resistant clones [73], allowing resistant strains to persist and spread within populations under antibiotic pressure. Additional mutations or rare compensatory changes in ribosomal proteins may also modulate resistance levels, although these are less well-characterized.

The MT28-Shanghai clone exemplifies the successful emergence and regional spread of macrolide-resistant *B. pertussis*. First identified in eastern China, this lineage has since been detected in multiple provinces and is associated with large-scale outbreaks in pediatric populations. Genomic analyses indicate that MT28-Shanghai is a descendant of the globally circulating ptxP3 lineage, demonstrating that antimicrobial resistance can emerge and converge with vaccine-driven adaptations, creating a high-fitness, multiadaptive clone [49]. The clone not only harbors the A2047G mutation but also exhibits the characteristic pertactin deficiency observed in many contemporary ptxP3 strains, highlighting the pathogen’s ability to accumulate multiple adaptive traits.

Other regions in Asia, such as Vietnam and Japan, have reported sporadic macrolide-resistant isolates, but these remain largely localized and less prevalent than in China. Resistance is extremely rare in Europe, North America, and Australia, suggesting that the selection and spread of resistant clones are closely linked to local antibiotic usage patterns. In China, for example, azithromycin and erythromycin are widely prescribed for both treatment and prophylaxis of respiratory infections, creating a strong selective environment for the emergence and maintenance of resistant lineages [74].

### 6.2. Molecular Epidemiology and Global Spread

Genomic studies have provided insights into the molecular epidemiology of macrolide-resistant *B. pertussis*. The MT28-Shanghai clone, for example, represents a single successful expansion of a resistant variant, rather than multiple independent emergence events, as suggested by phylogenetic analyses [75]. This highly fit, macrolide-resistant clone has become a dominant lineage in regions of East Asia. This finding indicates that once a fitness-neutral resistance mutation arises, it can rapidly disseminate in populations with high antibiotic pressure. Continued WGS-based surveillance is crucial to track the potential appearance of additional resistant lineages and to monitor any recombination events or compensatory mutations that might enhance their adaptability.

In summary, while macrolide-resistant *B. pertussis* remains largely confined to parts of Asia, the combination of high antibiotic use, global travel, and vaccine-driven strain adaptation creates conditions conducive to further spread. Understanding the molecular mechanisms of resistance, particularly the role of the A2047G mutation, and monitoring resistant clones through genomic surveillance are essential for maintaining effective pertussis treatment protocols and mitigating the public health impact of emerging resistant strains.

### 6.3. Implications for Treatment and Public Health

The emergence of macrolide-resistant *B. pertussis* carries several important clinical and public health implications. First, resistance compromises the efficacy of first-line therapy, potentially prolonging the infectious period and increasing the risk of transmission, particularly to unvaccinated infants [43]. Second, resistant strains may complicate post-exposure prophylaxis, which often relies on macrolides to prevent secondary cases in household and hospital settings. Third, the regional expansion of resistant lineages, such as MT28-Shanghai, underscores the need for enhanced genomic surveillance and antimicrobial susceptibility testing, particularly in regions with high macrolide consumption.

Alternative antibiotics, including trimethoprim-sulfamethoxazole, remain effective against resistant strains, but their use is limited in infants and certain patient populations due to safety concerns. The potential spread of macrolide resistance beyond Asia could pose a global threat if resistant clones are introduced into other regions, emphasizing the importance of integrated genomic and epidemiological monitoring. To mitigate this international risk, we recommend: (1) integrating macrolide minimum inhibitory concentration (MIC) testing into sentinel surveillance programs in countries with high volumes of international travel; and (2) considering trimethoprim-sulfamethoxazole (TMP-SMX) as an alternative for prophylaxis or treatment of suspected imported, resistant cases, in accordance with local guidelines.

In conclusion, while macrolide-resistant *B. pertussis* is mainly confined to several parts of Asia, the combination of increased use of antibiotics with international travel and vaccine-driven strain selection creates a conducive environment for further spread. It is imperative to understand the molecular mechanism of resistance, especially the role of A2047G mutation, and to continue genomic surveillance for resistant clones. These activities are essential to maintaining appropriate pertussis treatment paradigms and mitigating public health implications resulting from new resistance strains.

## 7. Epidemiological Consequences and Public Health Implications

The ongoing genomic evolution of *Bordetella pertussis* has profound implications for disease dynamics, age distribution, and public health strategies. Over the past two decades, the replacement of ancestral ptxP1 strains with ptxP3 lineages, combined with the emergence of PRN-isolates, has altered the epidemiological landscape of pertussis in both vaccinated and partially immune populations [20]. These genomic adaptations affect not only transmission efficiency but also the clinical and demographic patterns of disease.

One of the most notable consequences of these evolutionary changes is the shift in age-specific incidence. Historically, pertussis primarily affected infants and young children; however, contemporary epidemiological data reveal a significant increase in cases among adolescents and adults [76]. For example, in the prevaccine era, 85–90% of reported cases occurred in children between 1 and 10 years of age. Only 7–11% of the reported cases were recognized in infants, and reported adult cases were <3%. In the early whole-cell pertussis vaccine era, 54% of reported cases were noted in infants [77]. This trend correlates with the predominance of ptxP3 and PRN-deficient strains, which appear better adapted to infect partially immune hosts. Adolescents and adults, often experiencing mild or atypical symptoms, may unknowingly serve as reservoirs for transmission, facilitating the spread of infection to unvaccinated or incompletely vaccinated infants. Such shifts in age distribution complicate disease surveillance and outbreak control, as these older age groups are less likely to seek medical care or be clinically diagnosed with pertussis.

The evolution of *B. pertussis* is closely linked to vaccine-driven selection pressures. Acellular vaccines, which target a limited set of antigens, provide robust protection against severe disease but do not always prevent colonization or transmission [24]. Consequently, strains that modify or lose vaccine-targeted antigens, such as pertactin-deficient or ptxP3 variants, gain a selective advantage in highly immunized populations. The combination of waning immunity and antigenic divergence creates an ecological niche that facilitates the persistence and expansion of evolved strains. Epidemiological modeling indicates that these factors can contribute to cyclical epidemic waves every 3–5 years, driven by the accumulation of susceptible individuals, with estimated R_0_ values ranging from 5 to 17 [78], even in countries with high vaccination coverage [26].

In addition to affecting age distribution, genomic adaptations influence transmission dynamics and outbreak severity. The ptxP3 lineage has been associated with higher pertussis toxin expression, which may enhance bacterial shedding, prolong colonization, and increase secondary attack rates [79]. Although direct evidence linking specific lineages to clinical severity remains limited, observational studies suggest that the combination of higher toxin levels and immune evasion could increase the risk of symptomatic infection in partially immune hosts. This has implications for hospital preparedness, particularly in neonatal intensive care units where infants are most vulnerable.

From a public health perspective, these evolutionary changes necessitate a proactive update to control strategies. The key implications can be summarized as follows:

− Diagnosis: Increased clinical suspicion and PCR testing are needed for adolescents and adults presenting with prolonged cough.
− Treatment: In regions with potential importation of resistant strains, antibiotic susceptibility testing should be considered.
− Vaccination: Standard infant schedules are insufficient. Adolescent and adult booster doses, maternal immunization during pregnancy, and “cocooning” of newborns are essential to limit transmission.
− Surveillance: The non-negotiable need for WGS-integrated surveillance to track lineage replacement and antigenic variation in real-time.

Furthermore, ongoing genomic surveillance may inform the development of next-generation vaccines. These could include formulations that target conserved antigens less prone to mutation, live-attenuated vaccines capable of inducing mucosal immunity, or multi-epitope vaccines that provide broader protection against circulating variants.

Finally, the integration of genomic and epidemiological data allows public health authorities to anticipate emerging trends and tailor interventions accordingly. By tracking lineage replacement, antigenic variation, and antibiotic resistance, authorities can identify populations at heightened risk, adjust booster timing, and optimize vaccine formulations to maintain high levels of herd immunity. Such proactive measures are critical for sustaining pertussis control in the face of an evolving pathogen.

In summary, the genomic evolution of *B. pertussis* has reshaped the epidemiology of pertussis, altering age distribution, transmission patterns, and outbreak dynamics. Vaccine-driven selection, antigenic adaptation, and waning immunity collectively enable evolved strains to persist and spread, challenging conventional control strategies. Addressing these changes requires flexible vaccination policies, enhanced surveillance, and the development of vaccines capable of countering evolving lineages, ensuring continued protection against this highly contagious respiratory pathogen.

## 8. Challenges and Future Directions

### 8.1. Data and Surveillance Gaps

Effective control of *Bordetella pertussis* relies not only on vaccination and clinical management but also on robust surveillance systems that accurately capture pathogen evolution, circulation patterns, and emerging threats. While whole-genome sequencing (WGS) has revolutionized pertussis research, global genomic data remain unevenly distributed, with a heavy bias toward high-income countries [80]. Most published sequencing studies originate from Europe, North America, Japan, China, and Australia, leaving large regions of Africa, Latin America, and Southeast Asia underrepresented. This uneven coverage limits our ability to detect emerging lineages, track global transmission pathways, and anticipate shifts in strain prevalence that could influence vaccine effectiveness or resistance patterns.

In low- and middle-income countries, several barriers hinder comprehensive genomic surveillance. Limited laboratory infrastructure, scarcity of sequencing platforms, and insufficient bioinformatics expertise constrain the generation and analysis of genomic data [81]. In addition, inconsistent reporting and incomplete integration of clinical, vaccination, and epidemiological data reduce the usefulness of the genomic information that is available. Without high-quality, longitudinal datasets, it is challenging to assess the true prevalence of antigenic variants, monitor the emergence of macrolide-resistant clones, or evaluate the impact of vaccination programs on circulating strains.

Another critical gap lies in standardization and interoperability. Even where WGS data exist, differences in sequencing protocols, assembly pipelines, annotation methods, and metadata collection make cross-country comparisons difficult [82]. For example, some studies report only core-genome SNPs, while others include accessory genome analyses, complicating efforts to construct unified phylogenies or track global lineage replacement. Standardizing laboratory and bioinformatics approaches would enhance the comparability of datasets and enable more accurate global surveillance.

Data sharing and accessibility also remain challenges. While some genomic repositories, such as NCBI Pathogen Detection and the European Nucleotide Archive, facilitate open access, many datasets are not publicly available due to ethical, legal, or institutional constraints. Limited sharing slows the detection of emerging variants, including pertactin-deficient strains and macrolide-resistant clones, and reduces opportunities for coordinated global response [83]. Encouraging open-data policies and creating secure, privacy-compliant platforms for sharing genomic and epidemiological information are therefore essential.

Finally, integration of genomic data into routine public health decision-making is still limited in many countries. Sequencing often occurs retrospectively, as part of research studies, rather than in real-time to guide outbreak response, vaccination strategies, or antimicrobial stewardship. Expanding real-time surveillance systems that link sequencing with case reporting, demographic information, vaccination status, and antibiotic usage would greatly enhance the utility of genomic data for public health.

In summary, data and surveillance gaps represent a significant barrier to comprehensive pertussis control. Strengthening genomic infrastructure in underrepresented regions, standardizing sequencing and analysis protocols, and promoting open, integrated data sharing are critical steps to ensure early detection of emerging lineages, global monitoring of resistance, and informed vaccine policy decisions. Addressing these gaps is essential for anticipating the evolutionary trajectory of *B. pertussis* and maintaining effective, evidence-based public health interventions worldwide.

### 8.2. Vaccine Implications

The ongoing genomic evolution of *Bordetella pertussis* has important implications for vaccine design, effectiveness, and immunization strategies. Current acellular pertussis (aP) vaccines, which contain a limited number of antigens-typically pertussis toxin (Ptx), filamentous FHA, PRN, and FIM2/FIM3-have been highly effective in reducing severe disease and mortality. However, mounting evidence indicates that these vaccines provide incomplete protection against infection and transmission, particularly as circulating strains diverge antigenically from the vaccine components [21]. In addition to conventional component-purified aP vaccines, some countries have used co-purified acellular pertussis vaccines, in which pertussis antigens are co-purified and may include additional bacterial components beyond the canonical purified antigens [84,85]. Such formulations have been discussed as a potential “middle ground” between highly purified aP and wP vaccines, but comparative evidence on immunogenicity, durability, and real-world effectiveness across product types remains limited and context-dependent [84,86]. Explicit consideration of vaccine product heterogeneity (including co-purified formulations) may therefore be relevant when interpreting country-specific resurgence patterns and when designing next-generation vaccines. The predominance of ptxP3 and pertactin-deficient strains, combined with the emergence of antigenic variants in fimbrial and toxin genes, highlights the need for next-generation vaccines that account for the evolving pathogen landscape.

Short-term strategies should focus on optimizing the use of existing aP vaccines. This includes reinforcing adolescent and adult booster schedules, maximizing the coverage and effectiveness of maternal vaccination during pregnancy to protect newborns, and implementing ‘cocooning’ strategies around infants.

Mid-term strategies could involve a re-evaluation of wP vaccines in some contexts. Recent developments have led to wP vaccines with significantly reduced reactogenicity, making them a potentially viable option for certain populations where their broader immune response might be advantageous [87].

Long-term, the development of next-generation vaccines is paramount. Live-attenuated pertussis vaccines represent another promising avenue. Unlike acellular vaccines, live-attenuated formulations can induce robust mucosal and cellular immune responses, which are critical for preventing colonization and reducing transmission [88]. By stimulating both systemic and local immunity, these vaccines may overcome some of the limitations of current aP vaccines, particularly the inability to block asymptomatic carriage in adolescents and adults, who act as reservoirs for infant infections. Early-phase clinical trials of live-attenuated nasal vaccines have shown encouraging results in terms of safety, immunogenicity, and induction of mucosal IgA responses, suggesting potential for broader protective effects.

Booster strategies and schedule optimization are also critical components of vaccine policy in the era of genomic evolution. Evidence indicates that immunity conferred by acellular vaccines wanes significantly within 3–5 years, contributing to susceptibility among adolescents and adults. Tailored booster campaigns, including adolescent boosters, maternal vaccination during pregnancy, and cocooning strategies around newborns, can mitigate the risk of transmission to vulnerable populations. Integration of genomic surveillance into these strategies allows identification of the most prevalent and antigenically divergent strains, enabling vaccine schedules and formulations to be updated in a timely, evidence-based manner.

In addition, multi-epitope or recombinant subunit vaccines may provide enhanced protection by combining antigens from multiple circulating lineages. By incorporating multiple variants of pertactin, fimbrial proteins, and pertussis toxin, such vaccines could reduce immune escape and extend the duration of protection [89,90]. Computational modeling and reverse vaccinology approaches can identify candidate epitopes with high conservation and immunogenic potential, further guiding rational vaccine design in the context of pathogen evolution. Other innovative platforms, such as outer membrane vesicle (OMV) vaccines, are also under investigation.

Finally, the integration of vaccine development with global genomic surveillance is essential. Real-time monitoring of circulating lineages, antigenic variants, and antimicrobial resistance patterns can inform decisions regarding strain selection for updated vaccines, booster timing, and target populations. This approach ensures that vaccination strategies remain adaptive and responsive to the evolving epidemiology of pertussis, rather than relying solely on historical formulations. An ideal next-generation vaccine should aim to achieve three core objectives: (1) induce durable mucosal immunity to block colonization and transmission; (2) provide cross-protection against current and emerging variant strains, such as ptxP3 and PRN-; and (3) strike an optimal balance between the safety profile of aP and the broad, durable immunogenicity of wP.

### 8.3. Limitations

This review has several limitations. First, it is a narrative rather than a pre-registered systematic review, and we did not perform a formal risk-of-bias assessment or meta-analysis; the search, selection and synthesis of studies may therefore be incomplete. Second, genomic data from many low- and middle-income countries remain sparse, which constrains inference on global lineage dynamics and likely underestimates the diversity and spread of vaccine- and antibiotic-adapted strains. Third, we focus mainly on immunologic and evolutionary mechanisms; other drivers of pertussis epidemiology, including changes in diagnostics, surveillance practices, health care access and social behavior, are only briefly addressed. These limitations mean that the review should be read primarily as a framework for interpreting current evidence and for generating hypotheses and policy options, rather than as a definitive quantification of disease burden or intervention effects.

Further reading: Several recent reviews and commentaries provide complementary perspectives on pertussis resurgence, vaccine-induced immunity, and pathogen evolution, and may be useful for readers seeking broader background and alternative interpretations [17,22,63,68].

## 9. Conclusions

The global resurgence of pertussis in the acellular vaccine era reflects not a simple vaccine failure, but a dynamic interplay between waning host immunity, vaccine-driven pathogen evolution, and emerging antimicrobial resistance. Synthesizing epidemiological, immunological and genomic data, this review supports a three-driver framework in which: (i) imperfect and rapidly waning acellular vaccine induced immunity allows silent circulation of *Bordetella pertussis*; (ii) adaptive lineages such as ptxP3, frequently pertactin deficient, have swept through highly vaccinated populations; and (iii) macrolide resistant clones, exemplified by the MT28 Shanghai lineage, are beginning to undermine first line therapy in parts of Asia.

These converging processes have shifted the age distribution of disease towards adolescents and adults, increased the risk of transmission to unvaccinated infants, and complicated outbreak control. Sustainable control of pertussis will require evolutionary-informed strategies centered on three priorities: (1) integration of whole-genome sequencing into routine global surveillance to detect lineage replacement and resistance in real time; (2) development and deployment of next-generation vaccines that induce durable mucosal and cellular immunity and retain efficacy against contemporary variants; and (3) antibiotic stewardship policies that limit unnecessary macrolide use while preserving effective alternatives for treatment and prophylaxis.

Pertussis has therefore transitioned from a predominantly pediatric disease controlled by infant immunization to a re-emerging infection across all age groups, in which human interventions themselves shape pathogen evolution. Anticipating and counteracting this evolutionary response will be essential to ensure that advances in vaccinology and genomics translate into long-term, global control of whooping cough.

## Data Availability

No additional data has been generated for this review paper; all the necessary data are already presented in the manuscript.

## Abbreviations

The following abbreviations are used in this manuscript:

aP	acellular pertussis vaccine;
wP	whole-cell pertussis vaccine;
WGS	whole-genome sequencing;
PT	pertussis toxin;
FHA	filamentous hemagglutinin;
Prn	pertactin;
Fim	fimbriae;
Tdap	tetanus–diphtheria–acellular pertussis vaccine;
MLVA	multilocus variable-number tandem-repeat analysis;
PFGE	pulsed-field gel electrophoresis;
SNP	single-nucleotide polymorphism;
AMR	antimicrobial resistance;
MIC	minimum inhibitory concentration;
TMP-SMX	trimethoprim-sulfamethoxazole;
Th1/Th2/Th17	T helper 1/2/17;
IFN-γ	interferon-gamma;
IL	interleukin;
IgA/IgG	immunoglobulin A/G;
PCR	polymerase chain reaction;
LMICs	low- and middle-income countries;
OMV	outer membrane vesicle.
